# A Porous TiAl6V4 Implant Material for Medical Application

**DOI:** 10.1155/2014/904230

**Published:** 2014-10-16

**Authors:** Axel Deing, Bérengère Luthringer, Daniel Laipple, Thomas Ebel, Regine Willumeit

**Affiliations:** Institute of Materials Research, Helmholtz-Zentrum Geesthacht, Max-Planck-Straße 1, 21502 Geesthacht, Germany

## Abstract

Increased durability of permanent TiAl6V4 implants still remains a requirement for the patient's well-being. One way to achieve a better bone-material connection is to enable bone “ingrowth” into the implant. Therefore, a new porous TiAl6V4 material was produced via metal injection moulding (MIM). Specimens with four different porosities were produced using gas-atomised spherical TiAl6V4 with different powder particle diameters, namely, “Small” (<45 *μ*m), “Medium” (45–63 *μ*m), “Mix” (90% 125–180 *μ*m + 10% <45 *μ*m), and “Large” (125–180 *μ*m). Tensile tests, compression tests, and resonant ultrasound spectroscopy (RUS) were used to analyse mechanical properties. These tests revealed an increasing Young's modulus with decreasing porosity; that is, “Large” and “Mix” exhibit mechanical properties closer to bone than to bulk material. By applying X-ray tomography (3D volume) and optical metallographic methods (2D volume and dimensions) the pores were dissected. The pore analysis of the “Mix” and “Large” samples showed pore volumes between 29% and 34%, respectively, with pore diameters ranging up to 175 *μ*m and even above 200 *μ*m for “Large.” Material cytotoxicity on bone cell lines (SaOs-2 and MG-63) and primary cells (human bone-derived cells, HBDC) was studied by MTT assays and highlighted an increasing viability with higher porosity.

## 1. Introduction

Metallic biomaterials, like titanium (Ti) and its alloys, are widely used in medical applications. Excellent biocompatibility, high strength, and good corrosion resistance [1, 2] make titanium alloys a good material for orthopaedic and dental implants. Current applications are, for example, bone screws and nails, parts of artificial heart valves, spinal fusion devices, and total hip replacements [3]. Occurring shielding effects, bone atrophy, and sequentially implant loosening are disadvantages when Young's modulus of solid implants (TiAl6V4: 114 GPa) is much higher than the one of human cortical bone (10–30 GPa) [4, 5]. By reasons of population ageing and increasing popularity of extreme sports as well as costs of implant replacement (i.e., revision surgery), implant life time has to be increased and optimized. Obviously, materials with mechanical properties closer to the ones of human bone are necessary to develop long lasting implants. Here, increasing material porosity was the strategy chosen to decrease its rigidity. Another advantage of porous materials is the possibility of cell ingrowth for further stability of the osseointegrated implant [5, 6]. A direct relation between pore size and bone formation is assumed, since it provides surface and space for cell adhesion and bone ingrowth [7]. Also the pore interconnection (even below 100 *μ*m) provides the way for cell migration and allows for an efficient* in vivo* blood vessel formation [7, 8].

Several techniques have been developed to produce porous metal (e.g., chemical vapour deposition, space holder method [9], selective laser melting [10], and direct laser metal sintering [11]) and even applied to produce porous titanium [5, 12, 13]. Metal injection moulding (MIM) has the advantages of fast production of rather high amount of complex and net-shaped parts (i.e., decreased fabrication costs) and the potential to tailor material porosities by using different size of metal powder or adjusted sintering temperature [14].

Here, MIM technique was successfully applied to produce four kinds of porous TiAl6V4 specimens (namely, “Small,” “Medium,” “Mix,” and “Large”). The porosity was investigated by means of X-ray tomography (3D) and optical microscopy analysis (2D). Mechanical properties of each material were investigated via tensile, compression, and resonant ultrasound spectroscopy (RUS) tests and the obtained results were compared and discussed. Material biocompatibility was tested with two cell lines (SaOs-2, MG-63) and also with primary cells (human bone-derived cells, HBDC).

## 2. Materials and Methods

### 2.1. Material Part

#### 2.1.1. Sample Production

Four different samples were obtained using spherical, gas-atomized TiAl6V4 grade 23 powders of different size with oxygen content in chemical analysis <0.07% (TLS Technik Spezialpulver, Bitterfeld, Germany). The powder particle diameter ranges from <45 *μ*m to 180 *μ*m. Samples were named according to the particle diameter employed: “Small” (<45 *μ*m), “Medium” (45–63 *μ*m), “Large” (125–180 *μ*m), and “Mix” (90% 125–180 *μ*m + 10% <45 *μ*m). From each of these materials three types of specimens were prepared, using MIM: (1) “dog-bone-shaped” samples for tensile test (according to ISO 2740); (2) cylinder-shaped specimens for compression tests (cut from tensile test specimens); and (3) disc-shaped samples with a diameter of 10 mm and a height of 2 mm for biological tests. During manufacturing process each individual compression test sample could be prepared plane-parallel by feeding forward the saw; cutting at right angle was performed by visual judgement and therefore was not cogent perfect. Anyway, specimens were tested and show even higher strength compared to tensile tests and natural cortical bone (Table 1). For biological tests mirror-polished nonporous TiAl6V4 disc-shaped samples with a diameter of 10 mm were employed as reference specimen (cut from a round bar (F. W. Hempel Legierungsmetall GmbH & Co. KG, Oberhausen, Germany)), polished by conventional procedures followed by final manual polishing with a Struers oxide polish suspension (OPS) compound (Struers GmbH, Hannover-Garbsen, Germany).

#### 2.1.2. MIM Procedure

MIM process can be divided into four major steps: “feedstock fabrication,” “injection moulding,” “debinding,” and “sintering” [14].

*Feedstock Production*. Gas-atomised spherical TiAl6V4 alloy powders and binder components (mainly paraffin and polyethylene derivatives; Merck KGaA, Darmstadt, Germany, and Basell Polyolefine GmbH, Frankfurt, Germany) were blended for 2 h using a Z-blade kneader (FEMIX Misch-und Knettechnik GmbH, Waiblingen, Germany). The weight ratio between metal powder and binder was 9 : 1. The feedstock production and powder handling were carried out in a controlled argon atmosphere.

*Injection Moulding.* The tensile test samples with an average length of 89.35 ± 0.08 mm were moulded with a maximum injection pressure of 100 MPa at ~130°C, thanks to an ALLROUNDER 320 S (ARBURG GmbH + Co KG, Loßburg, Germany) injection press. Specimens for the compression tests were cut from the cylindrical part of tensile test samples with a ratio of 1 : 1.5 (diameter : height). The disc-shaped specimens were moulded with an MCP 100-KSA injection press (MCP HEK GmbH, Lübeck, Germany) with a pressure of 0.45 MPa for “Small” and “Medium,” 0.7 MPa for “Mix,” or 0.8 MPa for “Large.” Temperature of the injector was adjusted to 115°C (“Small” and “Medium”) or 120°C (“Mix” and “Large”). In the green state of the samples, the 30 mm disc-shaped specimens were cut into 10 mm discs with the help of a punch. 

*Debinding.* The chemical debinding of the samples was done for 20 h in hexane (Sigma-Aldrich Chemie GmbH, Munich, Germany) to extract the paraffin in a solvent debinding furnace (EBA 50, LÖMI GmbH, Aschaffenburg, Germany). Samples were then transferred to the sintering furnace (XVAC, XERION Ofentechnik GmbH, Freiberg, Germany) to perform the thermal debinding at 450°C and 600°C, each 1 h, under argon gas flow.

*Sintering.* All specimens were sintered at 1300°C for 2 h in a cold-wall furnace with molybdenum shieldings and tungsten heater under a vacuum of 10^−5^ mbar (XVAC, XERION Ofentechnik GmbH, Freiberg, Germany).

#### 2.1.3. Microstructure Characterization

Investigation of the pore size distribution, pore diameters, and the materials porosity was determined by material cross sectional microphotographs (optical microscopy (OM); Olympus PMG 3; Olympus, Hamburg, Germany) and further analysed by image analysis software (analySIS pro 5.0 (Olympus Soft Imaging Solutions GmbH, Münster, Germany) and Adobe-Photoshop CS5 software (Adobe Systems Incorporated, version 12.0.5x32)).

The materials pore volume was determined in two ways. First, the 2D pore volume was estimated via Photoshop software by calculating the ratio between black (pore) and white (material) pixels. The second pore volume analysis (3D) was performed using a Phoenix Nanotom X-ray tube tomography system equipped with a tungsten X-ray source (GE Sensing & Inspection Technologies GmbH, Hürth, Germany) in quadruplicates. Scanning parameters were set to 140 kV source voltage, 45 *μ*A (“Mix”) and 50 *μ*A (“Large”) source current, 0.5 mm copper filter, 1000 ms (“Large”) and 1250 ms (“Mix”) exposure time, and 2304 × 2304 pixel detector size. Image reconstruction was accomplished using the software DatosX Reconstruction 1.5 (GE Sensing & Inspection Technologies GmbH, Hürth, Germany). The compressed datasets (2x binning) of the specimens “Mix” and “Large” were used in this study. The voxel resolution was 12 *μ*m. This was too low to analyse the pore sizes in “Small” and “Medium.” For “Mix” and “Large” the samples' 3D pore volume was investigated using the software myVGL 2.1 (Volume Graphics GmbH, Heidelberg, Germany).

Scanning electron microscope (SEM) measurements were done by an Auriga 40 (Carl Zeiss, Oberkochen, Germany) at 3 kV accelerating voltage with the secondary-electrons detector and 3.5 mm working distance.

Conventional LECO melt extraction systems (TC-436AR and CS-444, LECO, Mönchengladbach, Germany) were used to determine the level of the interstitial elements oxygen and carbon.

#### 2.1.4. Mechanical Tests

The tensile tests were performed according to the standard test DIN EN ISO 6892-1, at room temperature at a strain rate of 1.2 × 10^−5^ s^−1^. For the test a servohydraulic structural test machine (Schenck, Zwick GmbH & Co. KG, Ulm, Germany) equipped with a 100 kN load cell was applied to at least 6 samples of each material. The compression tests (DIN 50106) were carried out under the same conditions using the same test machine as described for the tensile tests (*n* = 6). The determination of the shrinkage (*S*(%)) was done by comparing the samples length as green part (*L*_1_) and after sintering (*L*_2_):

(1) $$\begin{array}{c} S\left(\%\right)=\left(\frac{L_{1}-L_{2}}{L_{1}}\right)\times 100. \end{array}$$

Resonant ultrasound spectroscopy (RUS) tests were carried out in triplicates of each material at room temperature using the resonant frequency damping analyser (RFDA, IMCE, Genk, Belgium) professional according to the test standard ASTM E 1876. The RFDA software (IMCE, Genk, Belgium) calculates the elastic modulus via geometry, density, and frequency damping of the tested material.

#### 2.1.5. Cell Ingrowth

SaOs-2 cells were cultured for 2 days on “Mix” samples as described in Section 2.2.1. Samples were then critical-point-dried before scanning electronic microscope (SEM) evaluation (Auriga; Carl Zeiss, Jena, Germany). In brief, after a glutaraldehyde (Sigma-Aldrich Chemie GmbH, Munich, Germany) fixation step, carriers were stained in osmium tetroxide (Sigma-Aldrich Chemie GmbH, Munich, Germany) prior to an alcoholic dehydration row. Subsequently, samples were critical-point-dried in 2-propanol (Sigma-Aldrich Chemie GmbH, Munich, Germany) to preserve cell morphology by a Leica EM CPD300 (Leica Mikrosysteme Vertrieb GmbH, Wetzlar, Germany). Cells on carriers were then visualised by low voltage mode in charge contrast, using the SEM InLens detector. Samples were also broken in the middle in order to appreciate cell-sample colonisation along the cross section.

#### 2.1.6. Statistical Analysis

The results are presented as mean values ± standard deviation (Table 1, Figure 4). For the pore size distribution (Figure 3) the results were combined in 25 *μ*m steps and are displayed in clusters.

Statistics were performed using the SigmaStat software package (Systat software GmbH, Erkrath, Germany). Prior to statistical analysis data were analysed for normality and equal variance. Standard analysis comparing two treatments (for MTT assay; Figure 4(a)) was performed by using the* t*-test. Analysis of more than two treatments (for MTT/DNA; Figure 4(b)) was done by using the one-way analysis of variance (ANOVA; comparison against control group). Depending on the data distribution, either a one-way ANOVA or an ANOVA on ranks was performed. Post hoc tests were Bonferroni or Dunn's, respectively. Statistical values are indicated at the relevant experiments.

### 2.2. Biological Part

Prior to biological tests culture substrates were cleaned by immersing them in 2% Hellmanex solution (Hellma, Müllheim, Germany) and ultrasonication at room temperature for 20 min. These steps were repeated by replacing the Hellmanex solution with chloroform, then ethanol (Merck, Darmstadt, Germany), optional chloroform/methanol (80/20) (Merck, Darmstadt, Germany) to remove cell debris (if samples were used before), and finally ddH_2_O (Millipore, Billerica, Massachusetts, USA). Then samples were autoclaved for 20 min at 121°C (Systec VE-150, Systec GmbH, Wettenberg, Germany).

#### 2.2.1. Cell Culture

*Human Bone-Derived Cells (HBDC).* HBDC isolation was performed on bone splinters obtained from total hip replacement and approved by the local ethical committee [15, 16]. Adapted from Gartland et al. [16], cancellous bone pieces of about 5 mm were cut and cultured in Dulbecco's modified Eagle medium (DMEM) Glutamax-I (Invitrogen Corporation, Karlsruhe, Germany) with 10% foetal bovine serum (FBS, PAA Laboratories GmbH, Linz, Austria), 1% penicillin, and streptomycin (Invitrogen Corporation, Karlsruhe, Germany) for about 10 days without medium change. At visibility of outgrowing HBDC, the medium was changed every 3 days. Passaging was done at about 80% confluence. Cells in the 2nd passage were used to perform the experiments. 

*SaOs-2 and MG-63 Cells.* Human osteosarcoma cell lines SaOs-2 and MG-63 were obtained from the European collection of cell cultures (ECACC, Salisbury, UK). MG-63 and SaOs-2 cells were cultured in DMEM Glutamax-I with 10% FBS and McCoy's 5 A (Invitrogen Corporation, Karlsruhe, Germany) with 10% FBS, respectively. The medium was changed every 2-3 days. Passaging was done at about 80% confluence.

All cells were cultivated at 37°C under 5% CO_2_ and 95% humidity controlled atmosphere.

#### 2.2.2. Biological Tests

*MTT-Assay.* Metabolic activity was determined by the cell proliferation Kit MTT (Roche Diagnostics GmbH, Mannheim, Germany). The MTT assay is based on the cleavage of the yellow tetrazolium salt MTT (thiazolyl blue tetrazolium bromide) into purple formazan by metabolically active cells. HBDC, SaOs-2, or MG-63 cells were seeded on the different porous and mirror-polished (control) specimens in a density of 5 × 10^4^ cells/sample in 50 *μ*L medium in beforehand agarose coated 24-well plates. After 40 min adherence, 1 mL of cell specific medium was added. The cells were then further cultured for 1 or 3 days before addition of 100 *μ*L of MTT solution (5 mg/mL MTT in PBS). After an incubation period of 4 h the formed crystals were lysed by adding 1 mL solubilization solution (10% SDS in 0.01 M HCl) and incubated overnight in a humidified atmosphere at a temperature of 37°C and 5% CO_2_. The photometric quantification of the solubilized formazan product was performed using an ELISA reader (Tecan Sunrise, TECAN Deutschland GmbH, Crailsheim, Germany) at 570 nm with a reference wavelength of 655 nm.

*Quantitative Analysis of DNA.* This method, adapted from Labarca and Paigen [17], is based on the property of bisbenzimide fluorochrom to bind DNA. Samples were washed with phosphate-buffered saline (PBS) and immersed in 1 mL papain solution (10 mg/mL papain, Boehringer Mannheim GmbH, Mannheim, Germany; 5 *μ*L mercaptoethanol in 0.1 M NaH_2_PO_4_, VWR International GmbH, Darmstadt, Germany) at 60°C in order to digest the cells. The crude lysates were then incubated with 100 *μ*L of a bisbenzimide solution (2 M NaCl, 15 mM sodium citrate, and 2 *μ*g/mL bisbenzimide (Hoechst 33528; Serva Feinbiochemica GmbH & Co., Heidelberg, Germany) for 15 minutes in the dark and subsequently fluorometrically measured (excitation and emission wavelengths 355 nm and 460 nm, resp., with a VICTOR3 V. multilabel plate readers, Perkin Elmer, Rodgau-Juegesheim, Germany). The DNA concentrations were then obtained from a standard curve prepared with human genomic DNA (Sigma, Taufkirchen, Germany).

## 3. Results

### 3.1. Microstructure Analysis

**Figure 1:**
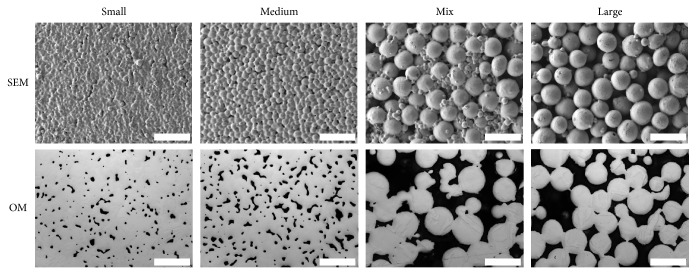
Material images. Scanning electron microscopy (SEM) and optical microscopy photographs (OM) of “Small,” “Medium,” “Mix,” and “Large” materials (scale bar 200 *μ*m).

Pictures of each material taken by SEM and optical microscopy are presented in Figure 1. Material distribution appears to be homogeneous for all four specimens.

**Figure 2:**
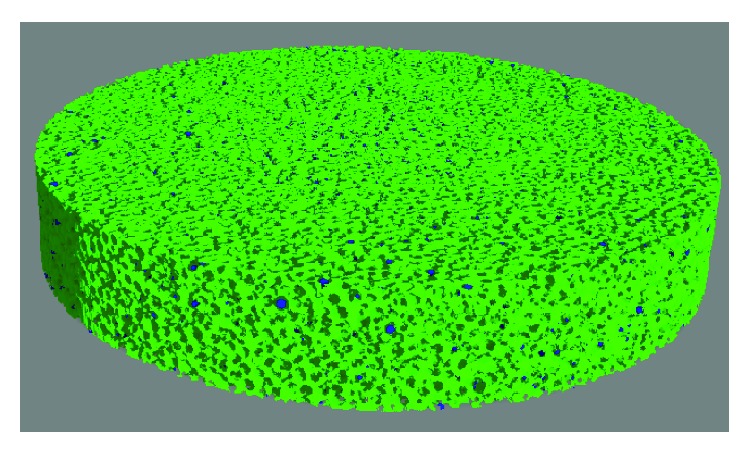
Microtomography. 3D image reconstruction of the pore volume of the specimen “Large” after measurement with X-ray microtomography. The dark areas represent the powder spheres while the pore volume is shown in green.

The 3D pore volume of “Mix” and “Large” (Figure 2) obtained via X-ray tomography was found to be 29.2 ± 0.6% and 34.1 ± 0.5% of the complete material volume for “Mix” and “Large,” respectively. In both materials this pore volume is formed by one interconnected pore. “Large” pore volume is 5% higher compared to “Mix.”

**Figure 3:**
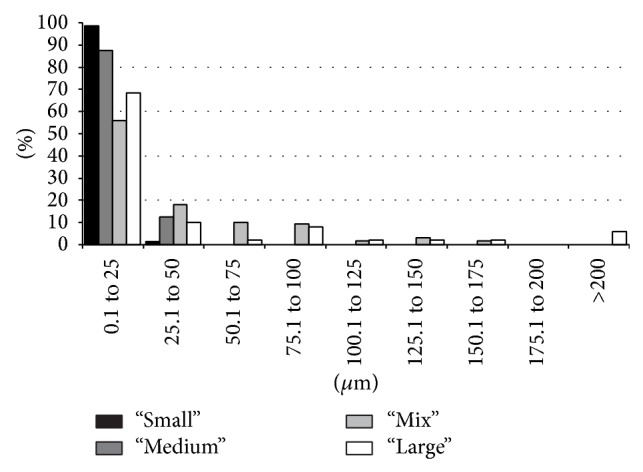
Pore sizes. Distribution of the pore sizes (in *μ*m) for each material (black, dark grey, light grey, and white for “Small,” “Medium,” “Mix,” and “Large,” resp.).

The 2D pore analysis from optical microscopy achieved pore volumes of 5 ± 1%, 11 ± 1%, 33 ± 5%, and 34 ± 1% for “Small,” “Medium,” “Mix,” and “Large,” respectively (see Table 1). The pore diameters were clustered in 25 *μ*m steps (see Figure 3). The calculated complete numbers of pores are 793, 806, 127, and 101 for “Small,” “Medium,” “Mix,” and “Large,” respectively. For the “Small” material nearly 100% of the pores are in the range between 0.1 and 25 *μ*m. 90% of “Medium” pore sizes are between 0.1 and 25 *μ*m and the remaining (10%) are between 25.1 and 50 *μ*m. “Mix” reveals a more dispersed pore size distribution. About 55% of the pores are up to 25 *μ*m in diameter, nearly 20% between 25.1 and 50 *μ*m and from 50.1 to 100 *μ*m it is 10% in each step. Between 100.1 and 175 *μ*m 5% of the pores can be observed in each 25 *μ*m cluster. The maximum for “Large” is up to 25 *μ*m with nearly 70%. The further pore size distribution can be compared to “Mix,” such that the ratio is twice decreased between 25.1 and 175 *μ*m. However, “Large” is the only material exhibiting pore diameters of more than 200 *μ*m.

### 3.2. Mechanical Properties

Tensile tests were performed in order to obtain Young's modulus (*E*), elongation to fracture (*ε*_*f*_), ultimate tensile strength (UTS), and yield strength (YS) for the four materials (presented in Table 1 in normal type). At least 6 specimens per material were tested. “Small” samples show Young's modulus of about 100 GPa, which is close to bulk material [23]. The “Medium” material has a little lower value of about 90 GPa, while Young's modulus value decreases extremely for “Mix” and “Large” (31 and 18 GPa, resp.). The elongations of “Small” and “Medium” reach up to 13.5% and 5.3%, whereas “Mix” and “Large” break already below 0.1%. “Small” and “Medium” show much higher UTS compared to “Mix” and “Large.” From compression tests, compressibility (**ε**_**c**_), ultimate compression strength (UCS), and compressive yield strength (CYS) were obtained from 6 samples of the same materials as mentioned above; the results are presented in Table 1 in bold. The compressibility for all materials is about 25% except for “Large” which is 14%. “Small” and “Medium” show UTS above 1300 MPa, whereas “Mix” and “Large” are below 700 MPa. The CYS of “Small” and “Medium” is higher than 700 MPa and for “Mix” and “Large” it is below 300 MPa. From resonant ultrasound spectroscopy (RUS) Young's modulus was achieved and is presented in Table 1 in italic. The results accord with the results obtained with tensile tests. Materials “Small” and “Medium” are above 92 GPa, whereas “Mix” and “Large” show results between 21 GPa and 42 GPa.

The sample shrinkage which occurs during the sintering process was examined by the difference in the axial dimension of the “dog-bone-shaped” specimens before and after this process. The green part length was detected to be 89.35 ± 0.08 mm for all materials. After sintering for 2 hours at 1300°C, the length of the samples changed differently for each material. “Small,” “Medium,” “Mix,” and “Large” showed percentage shrinkage of 11.8 ± 0.04%, 10.8 ± 0.05%, 4 ± 0.4%, and 2.9 ± 0.3%, respectively. The carbon content after the sintering process for all specimens was below 500 *μ*g per g. The oxygen content, presented in Table 1 with values between 1,500 and 2,000 *μ*g per g, is in the common range for this material [24]. The aforementioned values for oxygen and carbon content are typically observed after the MIM processing and do not reduce the ductility of these materials [25].

### 3.3. MTT Assay

MTT assays were carried out with two bone cell lines (SaOs-2 and MG-63) and with one bone-primary cell type (human bone-derived cells, HBDC) for each porous material (with *n* = 4) to study cell reactions (i.e., viability) either after one day or three days of culture. To have more accurate cell viability analyses the MTT absorbances were normalized by sample DNA content which is directly correlated with the number of adherent cells.

**Figure 4:**
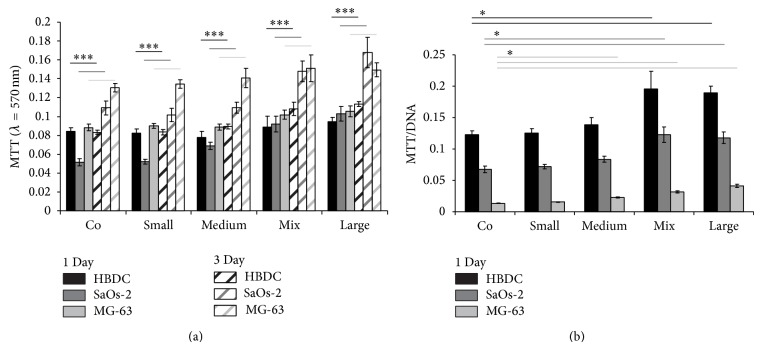
Viabilities. Three cell type MTT results per adherent cells: HBDC and osteosarcoma-derived cell lines SaOs-2 and MG-63. (a) The MTT assays were performed after 1 day (filled bars) and 3 days (striped bars) of culture. Significance levels were  ^***^ < 0.001. (b) The MTT results referred to the DNA content after 1 day. Significance levels were  ^*^ < 0.05.

Generally, a higher viability with increasing porosity can be observed (Figure 4). HBDC, SaOs-2, and MG-63 viabilities are also enhanced after 3 days of culture (Figure 4(a)). Normalization of the MTT results to the samples DNA content after 1 day results in increased values with raised porosity (Figure 4(b)).

### 3.4. Cell Ingrowth

**Figure 5:**
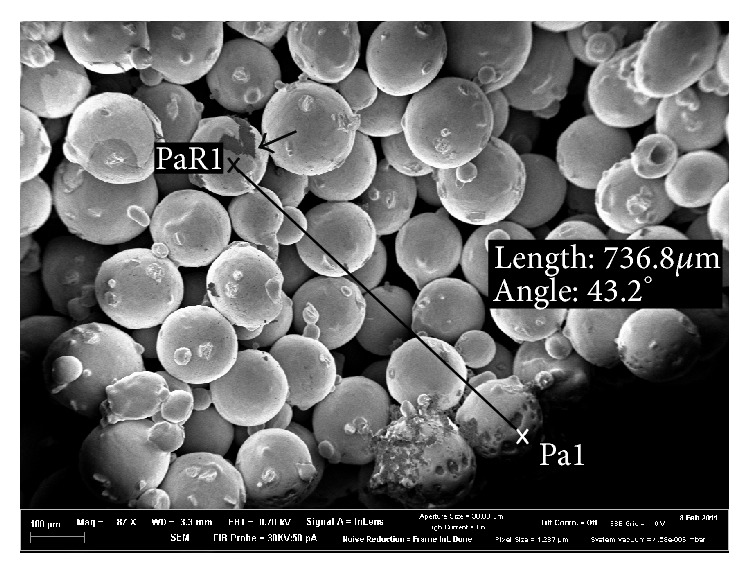
Cell ingrowth. SEM picture from the breaking edge of material “Mix” after 2 days of incubation with SaOs-2 cells. Pa1 is a point on the outer surface; point PaR1 is close to cells inside the material. Arrow points out the cells.

Investigation of cell ingrowth was performed in a first test by breaking a cell culture disc of material “Mix” and exploring the braking edge via SEM for cells. In Figure 5, cells are visible in about 735 *μ*m inside the material at an angle of 43.2°. Pa1 labels the point at the outer surface of the material. PaR1 is marking an area where cells could be observed.

## 4. Discussion

Metal injection moulding technology was successfully applied to produce four different porous materials with three different TiAl6V4 powder grain sizes. After studying their mechanical properties, cell-material reactions were examined.

The optical appearance of the freshly produced MIM parts as well as metallographic treated specimens is quite homogeneous (i.e., pore/metallic part distribution). The material “Mix” exhibits an increased Young's modulus (Table 1), compared to “Large,” due to a diminished pore volume fraction caused by the addition of the finer granulated powder. Young's moduli of about 18–21 GPa and 31–42 GPa for “Large” and “Mix,” respectively, are in a quite close range to that of bone (11–26 GPa) [23, 26]. Furthermore the ultimate tensile strength (UTS), yield strength (YS), and elongation values of “Mix” and “Large” are lower than the ones of “Small” and “Medium”; however, they are as well closer to the ones of bone (see Table 1) and possible additional bone ingrowth could further stabilize these materials. Therefore, a medical application of those materials seems to be beneficial to reduce stress-shielding effects observed for nonporous bulk titanium alloy implants [4, 5, 8], consequently preventing implant loosening. However, the materials “Small” and “Medium,” made from finer powder grain, result in Young's modulus close to that of bulk TiAl6V4 [23] and are therefore applicable in the field of conventional implementation. “Mix” and “Large” can be pertinent materials for less load-bearing implant applications such as smaller bone defect corrections.

The specimens for mechanical tests and cell culture tests were both produced with the same process (MIM) but with different machines. Mechanical tests rely on samples with standardised dimensions, but the dog-bone shaped samples cannot be used in cell culture. Therefore, it was necessary to use two different machines for sample production. However, the basic raw material (feedstock) was the same for all specimens. Because the binder powder ratio is chosen in such a way that just the space between the packed powder particles is filled, no significant difference in green density is possible, even if different moulding parameters or machines are applied. Because all the following processing steps (e.g., debinding and sintering) are absolutely the same for all specimens, biological and mechanical test samples reveal the same microstructure and porosity. If at all, only very little and not significant differences are possible.

It is highlighted by simulation that bone ingrowth in material with 50% interconnected porosity and pore sizes about 150–300 *μ*m will dramatically reduce stress shielding effects [27]. For this reason material porosity becomes more and more crucial in implant production. Additionally, quicker and more mature bone formation was obtained using a porous (70% porosity with 170 *μ*m mean pore size) rather than a solid structure [6]. It is pointed out* in vivo* that material porosity fraction plays a crucial role in tissue ingrowth, highlighting that a 30% porosity provides an excellent tissue ingrowth proofed by an increased calcium concentration within the pores [4]. Furthermore Bandyopadhyay et al. [4] accented the pore's interconnection to be fundamental. With X-ray tomography (3D analysis) pore volumes of 29% and 34% for “Mix” and “Large,” respectively, were shown as well as the interconnected pore network, consisting of just one single pore in these materials, thus fulfilling criteria presented above. The interconnection between the pores could be proofed by applying the abovementioned software after the tomography data was reconstructed. A criticism factor could be the tomography resolution of just about 12 *μ*m; this resulted in the distance of the sample between X-ray source and detector in the experimental setup. Anyway, this is an additional unusual method (3D) to the routinely used metallographic method (2D) for pore volume detection and the data shows good compliance (Table 1). An improvement would be the use of smaller samples during X-ray tomography, but for this study it was considered to be nonessential.

*In vitro* it was observed that all tested cell types show higher viability with higher porosity of the sample (Figure 4). The MTT results (Figure 4(a)) of HBDC and MG-63 after one day show similar results, whereas the values for SaOs-2 on materials control (“Co”) and “Small” are decreased. The viability increased for all tested cell types after 3 days, in case of SaOs-2 and MG-63 much stronger than for HBDC. This can be explained by the faster doubling time of the cell lines compared to primary cells (HBDC). To avoid the influence of cell growth rate, the results after 1 day culture were normalized with the DNA content on each specimen (Figure 4(b)). HBDC show the highest viability values compared to SaOs-2; MG-63 illustrates the lowest. This is suggestive of the highest compliance to this material for HBDC, then SaOs-2, and followed by MG-63. For all cells the viability values increase from material “Co” to “Large.”

As mentioned above the volume and interconnectivity of the pore network should be suitable for cell ingrowth. “Small” and “Medium” exhibit pore diameters (analysed from 2D optical microscopy pictures) up to 50 *μ*m without interconnected pores. “Mix” and “Large” on the other hand show pore diameters up to 175 *μ*m and for “Large” some pores are even bigger than 200 *μ*m (see Figure 3). The highest value for pore diameter is 0.1–25 *μ*m for “Mix” and for “Large.” But in contrast to “Small” and “Medium,” “Mix” and “Large” show pore diameters between 50 and 175 *μ*m as well. In “Large,” for example, about 22% of the pores are bigger than 50 *μ*m. The size of HBDC, which are one of the most important cells in osseointegration, is known to be between 20 and 30 *μ*m in diameter with a varying cell shape and elongated cell processes when attached [28]. Additionally, it has already been shown that bone ingrowth is possible in materials with a mean pore size of 100–300 *μ*m [29]. Furthermore the connections between the pores of porous biomaterials are an important pathway between the pores [7, 29] and recommended to be at least 20–40 *μ*m* in vitro* and 20–50 *μ*m* in vivo* for cell penetration [29]. In Figure 1 the SEM pictures of “Mix” and “Large” already illustrate the open porous structure of these scaffolds. Furthermore, the high interconnectivity was proven by tomography. It was already shown that cells can colonize materials with 200 *μ*m pore diameter [30], and even if the results of this study show a high amount of small pores below 25 *μ*m, the ratio of pore volume is mainly influenced by the pores with large volume. Additionally, due to the polishing process to obtain the 2D pictures of the materials, apparent small pores can be obtained (i.e., it depends on at which plane the polishing process is intersecting the pore (“sphere”): at lower and upper levels, diameters will be smaller). Furthermore, the numbers of smaller and bigger pores are gradually decreasing and increasing, respectively, for material with increased powder particle sizes. Therefore, cell ingrowth for “Mix” and “Large” is expected and demonstrated in a preliminary test. Cell ingrowth appeared to reach the middle of material “Mix” in cell culture samples (Figure 5). Further* in vitro* studies are still under investigation. The recommended pore sizes are a controversy discussed topic and* in vivo* tests would be great evidence.

## 5. Conclusions

The results of this study show that porous TiAl6V4 processed by MIM could be a well-suited net-shaped material for medical application. A material with a completely interconnected pore volume of about 30% and pore diameters up to 175 *μ*m could be suitable for osseointegration. This could prevent stress shielding in two ways: the mechanical properties are closer to that of bone and the connection with the tissue could take place “under” the visible surface. We assume that the porous materials could lead to less revision surgery, but* in vivo *tests should be the next strategy.

## Figures and Tables

**Table 1:** Material properties, mechanical properties, pore volumes, and oxygen contents of the different porous TiAl6V4 materials. The results of tensile, compression, and resonant ultrasound spectroscopy (RUS) tests are displayed in Roman, bold, and in italic fonts, respectively.

Material	Porosity		Mechanical properties				O_2_ content [*µ*g/g]
	Porosity 2D [%]	Porosity 3D [%]	*E* [GPa]	UTS (**UCS**) [MPa]	YS (**CYS**) [MPa]	*ε*_*f*_ (**ε**_**c**_) [%]	
“Small”	5 ± 1	—	101 ± 5	806 ± 2	707 ± 4	14 ± 2	1509
			*109 ± 3 *	**1358 ± 37**	**783 ± 16**	**26 ± 1**	

“Medium”	11 ± 1	—	93 ± 5	733 ± 24	628 ± 13	5 ± 3	2013
			*92 ± 3 *	**1341 ± 66**	**741 ± 18**	**26 ± 2**	

“Mix”	33 ± 5	29 ± 1	31 ± 6	95 ± 40	—	—	1918
			*42 ± 2 *	**623 ± 20**	**261 ± 10**	**25 ± 1**	

“Large”	34 ± 1	34 ± 1	18 ± 1	98 ± 10	—	—	1918
			*21 ± 2 *	**306 ± 7**	**152 ± 5**	**14 ± 1**	

Natural bone	—	—	10–30^a^	133^c^	—	—	—
			*8–33* ^b^	176^d^	130–180^e^		

^a^[4, 5, 18]; ^b^[19, 20]; ^c^[21]; ^d^[22]; ^e^[18].

CYS: compressive yield strength, *E*: Young's modulus, *ε*_*f*_: elongation to fracture, **ε**_**c**_: compressibility, UCS: ultimate compression strength, UTS: ultimate tensile strength, and YS: yield strength.

## References

[1] Long M., Rack H. J. (1998). Titanium alloys in total joint replacement—a materials science perspective. *Biomaterials*.

[2] Okazaki Y., Rao S., Ito Y., Tateishi T. (1998). Corrosion resistance, mechanical properties: corrosion fatigue strength and cytocompatibility of new Ti alloys without A1 and V. *Biomaterials*.

[3] Ebel T. (2008). Titanium and titanium alloys for medical applications: opportunities and challenges. *PIM International*.

[4] Bandyopadhyay A., Espana F., Balla V. K., Bose S., Ohgami Y., Davies N. M. (2010). Influence of porosity on mechanical properties and *in vivo* response of Ti6Al4V implants. *Acta Biomaterialia*.

[5] Spoerke E. D., Murray N. G., Li H., Brinson L. C., Dunand D. C., Stupp S. I. (2005). A bioactive titanium foam scaffold for bone repair. *Acta Biomaterialia*.

[6] Chang Y. S., Oka M., Kobayashi M., Gu H.-O., Li Z.-L., Nakamura T., Ikada Y. (1996). Significance of interstitial bone ingrowth under load-bearing conditions: a comparison between solid and porous implant materials. *Biomaterials*.

[7] Mastrogiacomo M., Scaglione S., Martinetti R., Dolcini L., Beltrame F., Cancedda R., Quarto R. (2006). Role of scaffold internal structure on *in vivo* bone formation in macroporous calcium phosphate bioceramics. *Biomaterials*.

[8] Kuboki Y., Takita H., Kobayashi D., Tsuruga E., Inoue M., Murata M. (1998). BMP-induced osteogenesis on the surface of hydroxyapatite with geometrically feasible and nonfeasible structures: topology of osteogenesis. *Journal of Biomedical Materials Research*.

[9] Ryan G., Pandit A., Apatsidis D. P. (2006). Fabrication methods of porous metals for use in orthopaedic applications. *Biomaterials*.

[10] Mullen L., Stamp R. C., Brooks W. K., Jones E., Sutcliffe C. J. (2009). Selective laser melting: a regular unit cell approach for the manufacture of porous, titanium, bone in-growth constructs, suitable for orthopedic applications. *Journal of Biomedical Materials Research B: Applied Biomaterials*.

[11] Traini T., Mangano C., Sammons R. L., Mangano F., Macchi A., Piattelli A. (2008). Direct laser metal sintering as a new approach to fabrication of an isoelastic functionally graded material for manufacture of porous titanium dental implants. *Dental Materials*.

[12] Wen C. E., Yamada Y., Shimojima K., Chino Y., Hosokawa H., Mabuchi M. (2002). Novel titanium foam for bone tissue engineering. *Journal of Materials Research*.

[13] Shen H., Brinson L. C. (2011). A numerical investigation of porous titanium as orthopedic implant material. *Mechanics of Materials*.

[14] Aust E., Limberg W., Gerling R., Oger B., Ebel T. (2006). Advanced TiAl6Nb7 bone screw implant fabricated by metal injection moulding. *Advanced Engineering Materials*.

[15] Gallagher J. A. (2003). Human osteoblast culture. *Methods in Molecular Medicine*.

[16] Gartland A., Rumney R. M. H., Dillon J. P., Gallagher J. A. (2012). Isolation and culture of human osteoblasts. *Methods in Molecular Biology*.

[17] Labarca C., Paigen K. (1980). A simple, rapid, and sensitive DNA assay procedure. *Analytical Biochemistry*.

[18] Staiger M. P., Pietak A. M., Huadmai J., Dias G. (2006). Magnesium and its alloys as orthopedic biomaterials: a review. *Biomaterials*.

[19] Yoon H. S., Katz J. L. (1976). Ultrasonic wave propagation in human cortical bone. II. Measurements of elastic properties and microhardness. *Journal of Biomechanics*.

[20] Lee T., Lakes R. S., Lal A. (2002). Investigation of bovine bone by resonant ultrasound spectroscopy and transmission ultrasound. *Biomechanics and Modeling in Mechanobiology*.

[21] Turner C. H., Wang T., Burr D. B. (2001). Shear strength and fatigue properties of human cortical bone determined from pure shear tests. *Calcified Tissue International*.

[22] Schmidt-Nielsen K. (1984). *Scaling: Why is Animal Size so Important?*.

[23] Niinomi M. (2002). Recent metallic materials for biomedical applications. *Metallurgical and Materials Transactions A*.

[24] Ferri O. M., Ebel T., Bormann R. (2010). Influence of surface quality and porosity on fatigue behaviour of Ti-6Al-4V components processed by MIM. *Materials Science and Engineering A*.

[25] Ebel T., Ferri O. M., Limberg W., Schimansky F. P. (2011). Metal injection moulding of advanced titanium alloys. *Advances in Powder Metallurgy & Particulate Materials*.

[26] Weiner S., Wagner H. D. (1998). The material bone: structure-mechanical function relations. *Annual Review of Materials Science*.

[27] Li H., Oppenheimer S. M., Stupp S. I., Dunand D. C., Brinson L. C. (2004). Effects of pore morphology and bone ingrowth on mechanical properties of microporous titanium as an orthopaedic implant material. *Materials Transactions*.

[28] Pautke C., Schieker M., Tischer T., Kolk A., Neth P., Mutschler W., Milz S. (2004). Characterization of osteosarcoma cell lines MG-63, Saos-2 and U-2 OS in comparison to human osteoblasts. *Anticancer Research*.

[29] Lu J. X., Flautre B., Anselme K., Hardouin P., Gallur A., Descamps M., Thierry B. (1999). Role of interconnections in porous bioceramics on bone recolonization *n vitro* and *in vivo*. *Journal of Materials Science: Materials in Medicine*.

[30] Pamula E., Bacakova L., Filova E., Buczynska J., Dobrzynski P., Noskova L., Grausova L. (2008). The influence of pore size on colonization of poly(L-lactide-glycolide) scaffolds with human osteoblast-like MG 63 cells *n vitro*. *Journal of Materials Science: Materials in Medicine*.

